# Supplementary material for: Staphylococcal phages and pathogenicity islands drive plasmid evolution
Source: Nat Commun. 2021 Oct 6;12:5845. doi: 10.1038/s41467-021-26101-5 (PMC8494744; doi:10.1038/s41467-021-26101-5)
Supplement: Supplementary file 1 — Supplementary Information [file 41467_2021_26101_MOESM1_ESM.pdf]

Supplementary Table 1: Phage and SaPI titres

Background		pC221		pI258		pGO1	
		Phage titre[a]	SaPI titre[b]	Phage titre	SaPI titre	Phage titre	SaPI titre
80α		10.02 (±0.21)	-	9.74 (±0.04)	-	9.96 (±0.05)	-
80α Δ*ter*S		<1[c]	-	<1	-	ND[d]	-
80α::SaPI1*cpm*AB		<1	-	<1	-	ND	-
80α	SaPI1	8.40 (±0.21)	7.98 (±0.37)	8.55 (±0.21)	7.86 (±0.30)	8.28 (±0.22)	7.64 (±0.38)
80α Δ*ter*S	SaPI1	<1	7.99 (±0.51)	<1	8.10 (±0.70)	ND	ND
80α	SaPI1Δ*ter*S	8.55 (±0.32)	5.89 (±0.17)	9.06 (±0.46)	5.75 (±0.23)	ND	ND
80α Δ*ter*S	SaPI1Δ*ter*S	<1	<1	<1	<1	ND	ND
80α	SaPI1Δ*cpm*AB	9.88 (±0.13)	9.06 (±0.04)	9.66 (±0.12)	8.74 (±0.25)	9.75 (±0.37)	8.83 (±0.31)
80α::SaPI1*cpm*AB	SaPI1	<1	7.01 (±0.21)	<1	6.86 (±0.35)	ND	ND
80α	SaPIbov2	8.25 (±0.42)	7.79 (±0.18)	8.08 (±0.10)	8.03 (±0.22)	8.58 (±0.18)	7.83 (±0.12)

[a]Mean log_{10} Plaque-forming units (Pfu) per ml of lysate in RN4220 recipient, n = 3 (± std).
[b]Mean log_{10} Transduction units (TrU) per ml of lysate in RN4220 recipient, n = 3 (± std).
[c]Limit of detection = 1.
[d]ND, Not determined.

Supplementary Table 2: Transfer abilities of the evolved pGO1 plasmids.

Plasmid	Size (bp)	Potential for conjugative transfer[a]	Median conjugation efficiency (range)[b,c]	Potential for transduction (< 45kb)	Mean Log_{10} TrU per ml (±SD, n=3) [d]	Median transduction efficiency (range) [e,f]
pGO1	54,000	Yes (tra^+, nes^+, $oriT^+$)	4.42×10^{-4} (3.85×10^{-5} to 5.96×10^{-4})	No	<1	$< 1.00 \times 10^{-8}$
pGO1evol	27,574	No (tra^-, nes^+ $oriT^+$)	$< 7.5 \times 10^{-9}$	Yes	1.32 (± 0.28)	3.00×10^{-8} (1.00×10^{-8} to 3.00×10^{-8})
$pGO1evol_A$*	41,026	Yes (tra^+, nes^+ $oriT^+$)	3.55×10^{-3} (1.50×10^{-3} to 1.55×10^{-2})	Yes	2.23 (± 0.26)	1.80×10^{-7} (9.00×10^{-8} to 3.00×10^{-7})
$pGO1evol_B$*	41,026	Yes (tra^+, nes^+ $oriT^+$)	ND	Yes	2.42 (± 0.09)	2.80×10^{-7} (2.10×10^{-7} to 3.10×10^{-7})
$pGO1evol_C$*‡	40,932	No (tra^-, nes^- $oriT^-$)	$< 7.5 \times 10^{-9}$	Yes	5.03 (± 0.13)	1.12×10^{-3} (7.80×10^{-4} to 1.41×10^{-3})
$pGO1evol_F$*	41,737	Yes (tra^+, nes^+ $oriT^+$)	2.63×10^{-3} (5.65×10^{-5} to 4.97×10^{-3})	Yes	2.91 (± 0.03)	8.20×10^{-7} (7.50×10^{-7} to 8.80×10^{-7})
$pGO1evol_J$*‡	43,843	No (tra^+, nes^- $oriT^-$)	$< 7.5 \times 10^{-9}$	Yes	7.04 (± 0.13)	9.80×10^{-2} (8.90×10^{-2} to 1.53×10^{-1})
$pGO1evol_E$†	30,899	No (tra^-, nes^+ $oriT^+$)	$< 7.5 \times 10^{-9}$	Yes	1.16 (± 0.28)	1.00×10^{-8} (1.00×10^{-8} to 3.00×10^{-8})
$pGO1evol_H$†	30,899	No (tra^-, nes^+ $oriT^+$)	ND	Yes	0.33 (± 0.58)	$<1.00 \times 10^{-8}$ ($<1.00 \times 10^{-8}$ to 1.00×10^{-8})
$pGO1evol_I$†	30,899	No (tra^-, nes^+ $oriT^+$)	$< 7.5 \times 10^{-9}$	Yes	1.80 (± 0.27)	5.00×10^{-8} (4.00×10^{-8} to 1.30×10^{-7})

ND: Not determined; [a] The products of the conjugative transfer cluster genes (*tra*), relaxase (*nes*) and origin of transfer (*ori*T) are necessary for mating pore formation and translocation of plasmid DNA between cells; [b] Gentamicin-resistant transconjugant cells/donor cells, n = 3; [c] No transfer was considered to be $< 7.5 \times 10^{-9}$; [d] Transduction of 100µl of lysate into 1ml of RN4220 recipient cells; [e] Gentamicin-resistant transductant cells per transducing particle following infection of RN4220 recipient cells with 10^8 transducing particles (10^7 transducing particles where SaPIbov2 is present), n = 3; [f] No transfer was considered to be $< 1.0 \times 10^{-8}$; *SaPIbov2-driven remodelling; †SaPI1Δ*cpm*AB-driven remodelling; ‡pGO1-SaPIbov2 hybrid species.

Supplementary Table 3: Size reduction of pGO1evol forces a trade-off between mechanisms for mobilization.

Mating				Distribution of pGO1 and pGO1evol plasmids in transconjugant populations				
Donor 1	Donor 2	Recipient	Median conjugation efficiency (range) [a]	Neomycin-resistant colonies [b] Exp't 1	Exp't 2	Exp't 3	Frequency of pGO1 transfer (%)	Frequency of pGO1evol$_X$ transfer (%)
SH1000 pGO1	SH1000 pGO1evol	RN4220	2.98×10^{-5} (3.16×10^{-6} to 1.04×10^{-4})	100/100	100/100	100/100	100.0 (± 0.00)	0.00 (± 0.00)
SH1000 pGO1	SH1000 pGO1evol$_A$	RN4220	9.83×10^{-4} (6.56×10^{-4} to 6.29×10^{-3})	19/100	9/100	33/100	20.3 (± 12.1)	79.7 (± 12.1)
SH1000 pGO1	SH1000 pGO1evol$_F$	RN4220	8.36×10^{-4} (6.05×10^{-4} to 1.22×10^{-3})	100/100	100/100	100/100	100.0 (± 0.00)	0.00 (± 0.00)
SH1000 pGO1	SH1000 pGO1evol$_E$	RN4220	2.12×10^{-4} (4.16×10^{-5} to 2.30×10^{-4})	100/100	100/100	100/100	100.0 (± 0.00)	0.00 (± 0.00)
SH1000 pGO1	SH1000 pGO1evol$_I$	RN4220	1.60×10^{-6} (1.17×10^{-6} to 1.69×10^{-4})	34/34	43/43	100/100	100.0 (± 0.00)	0.00 (± 0.00)

[a] Three independent mating experiments were carried out for each donor combination; [b] Neomycin resistance was used to differentiate pGO1 from the derivative plasmids as all pGO1evol$_X$ plasmids are sensitive to neomycin.

Supplementary Table 4: Bacterial strains and plasmids

Strain	Description	Reference
RN450	*Staphylococcus aureus* NCTC8325 cured of ϕ11, ϕ12 and ϕ13	[1]
RN4220	Restriction-deficient derivative of RN450	Lab strain
SH1000	*rsb*U$^+$ derivative of *S. aureus* NCTC8325-4	Lab strain
RN10359	RN450 80α	[2]
SH1000 pGO1	SH1000 pGO1	[3]
JP20320	SH1000 80α	This study
JP19979	SH1000 pGO1 80α	This study
JP19994	JP19979 SaPI1 *tst*::*tet*M	This study
JP19996	JP19979 SaPIbov2 *bap*::*tet*M	This study
JP21467	RN4220 80α pGO1evol$_A$	This study
JP21468	RN4220 80α pGO1evol$_B$	This study
JP21469	RN4220 pGO1evol$_C$::SaPIbov2 *bap*::*tet*M	This study
JP21470	RN4220 pGO1evol$_F$	This study
JP21471	RN4220 pGO1evol$_J$::SaPIbov2 *bap*::*tet*M	This study
JP21472	RN4220 pGO1evol$_E$	This study
JP21473	RN4220 pGO1evol$_H$	This study
JP21474	RN4220 pGO1evol$_I$	This study
JP19881	RN4220 80α pGO1evol	This study
JP20000	SH1000 pGO1evol	This study
JP20050	JP20320 pGO1evol	This study
JP21271	SH1000 pGO1evol$_A$	This study
JP21272	SH1000 pGO1evol$_C$::SaPIbov2 *bap*::*tet*M	This study
JP21273	SH1000 pGO1evol$_F$	This study
JP21274	SH1000 pGO1evol$_J$::SaPIbov2 *bap*::*tet*M	This study
JP21275	SH1000 pGO1evol$_E$	This study
JP21276	SH1000 pGO1evol$_I$	This study
JP14151	RN4220 SAOUHSC 00390::*cad*CA; Cd^R marker 10 kb downstream SaPI type II *att*B	This study
JP19998	JP14151, spontaneous rifampicin-resistant mutant	This study
JP20580	JP20320 pGO1evol$_A$	This study
JP20581	JP20320 pGO1evol$_B$	This study
JP20582	JP20320 pGO1evol$_C$::SaPIbov2 *bap*::*tet*M	This study
JP20585	JP20320 pGO1evol$_E$	This study
JP20583	JP20320 pGO1evol$_F$	This study
JP20586	JP20320 pGO1evol$_H$	This study
JP20587	JP20320 pGO1evol$_I$	This study
JP20584	JP20320 pGO1evol$_J$::SaPIbov2 *bap*::*tet*M	This study
ST126	RN4220 80α SaPI1 *tst*::*tet*M Δ*cpm*AB	[4]

JP19997	JP20320 SaPI1 *tst*::*tet*M Δ*cpm*AB	This study
JP18286	RN4220 pI258	[5]
JP18292	JP18286 80α	This study
JP18410	JP18292 SaPI1 *tst*::*tet*M	This study
JP18413	JP18292 SaPIbov2 *bap*::*tet*M	This study
JP12871	RN4220 80α Δ*ter*S	This study
JP18208	JP12871 pI258	This study
JP18661	JP18208 SaPI1 *tst*::*tet*M	This study
JP18693	JP18292 SaPI1 *tst*::*tet*M Δ*ter*S	This study
JP18694	JP18208 SaPI1 *tst*::*tet*M Δ*ter*S	This study
JP18525	JP18292 SaPI1 *tst*::*tet*M Δ*cpm*AB	This study
ST82	RN4220 80α::SaPI1*cpm*AB	[4]
JP18669	ST82 pI258	This study
JP18692	JP18669 SaPI1 *tst*::*tet*M	This study
JP18288	RN4220 pC221	[6]
JP18293	JP18288 80α	This study
JP18665	JP18293 SaPI1 *tst*::*tet*M Δ*cpm*AB	This study
JP18406	JP18293 SaPI1 *tst*::*tet*M	This study
JP18408	JP18293 SaPIbov2 *bap*::*tet*M	This study
JP18425	JP12871 pC221	This study
JP18865	JP18425 SaPI1 *tst*::*tet*M	This study
JP18866	JP18293 SaPI1 *tst*::*tet*M Δ*ter*S	This study
JP18867	JP18425 SaPI1 *tst*::*tet*M Δ*ter*S	This study
JP19946	ST82 pC221	This study
JP19950	JP19946 SaPI1 *tst*::*tet*M	This study
JP1220	*Staphylococcus xylosus*	Lab strain
JP831	*Staphylococcus epidermidis* BM96184	Lab strain
JP7422	*Listeria monocytogenes* SK1351	Lab strain
JP21894	RN4220, spontaneous rifampicin-resistant mutant	This study
JP21480	RN450 pC221	This study
JP21924	JP21480 SaPI1 *tst*::*tet*M	This study
JP21481	RN10359 pC221	This study
JP21925	JP21481 SaPI1 *tst*::*tet*M	This study

Plasmid	Description	Reference
pGO1	*S. aureus* conjugative plasmid, 54 kb; confers resistance to bleomycin, gentamicin, kanamycin, neomycin, quaternary ammonium compounds, and trimethoprim	[3]
pI258	*S. aureus* plasmid, 29 kb; confers resistance to erythromycin, cadmium, arsenic, and mercury	[7]
pC221	*S. aureus* mobilizable plasmid, 4.6 kb; confers resistance to chloramphenicol	[6]

Supplementary Table 5: Oligonucleotides

Primer	Sequence (5'-3')	Description
Differentiation of pGO1 and pGO1evolved plasmids		
pGO1_p01-F	GATAACAACAAAAACGAATAATATAGGAGGGGTT	Region common to both pGO1 and pGO1-derivatives
pGO1_p01-R	GTTACCCCACCTCTTTAATACTGTAGGCTA	
pGO1-1F	CACTCAACTATTATGAGAAGAAAAAATATGGAAAACAATCCA	PCR for differentiation of pGO1 and pGO1evol
pGO1_42317-R	GTCTTGATAGTTAGGTAACATTCCTATTGAGTG	
pGO1-Frag21F	TTGCAATATTGAAAGGAACACCTAAAAATATATCAGCAC	PCR for differentiation of pGO1 and pGO1evol$_A$
pGO1-Frag21R	TTTCTCAGCATAATTAATGGCCCGTGATG	
Southern blot DNA probes		
pGO1_GmR-F	AGAGCCAAATTATTGGAGTAAAGGAATTGG	*aac*A-*aph*D (gentamicin-resistance) probe
pGO1_GmR-R	TATCTGTATAATCTAAACCGTGCATTTGTC	
pGO1_nes-F	TTTGTGGAATAAAGTTCATGATGTTGAGAACAGA	*nes* probe
pGO1_Frag4R	TTCATCTTCAAATTTTCGTGCTTTCCAACC	
tetM-1mS	ACGCGTCGACCAAATATGCTCTTACGTGC	*tet*M (SaPIbov2 *bap::tet*M) probe
REV tetM mid	CATCAACATGAGCTAAAACTCCAA	

References

1. Novick, R. Properties of a cryptic high-frequency transducing phage in *Staphylococcus aureus*. *Virology* **33,** 155–166 (1967).
2. Christie, G. E. *et al.* The complete genomes of *Staphylococcus aureus* bacteriophages 80 and 80α--implications for the specificity of SaPI mobilization. *Virology* **407,** 381–390 (2010).
3. Caryl, J. A. & O'Neill, A. J. Complete nucleotide sequence of pGO1, the prototype conjugative plasmid from the Staphylococci. *Plasmid* **62,** 35–38 (2009).
4. Damle, P. K. *et al.* The roles of SaPI1 proteins gp7 (CpmA) and gp6 (CpmB) in capsid size determination and helper phage interference. *Virology* **432,** 277–282 (2012).
5. Wang, P. Z. & Novick, R. P. Nucleotide sequence and expression of the beta-lactamase gene from *Staphylococcus aureus* plasmid pI258 in Escherichia coli, Bacillus subtilis, and Staphylococcus aureus. *J. Bacteriol.* **169,** 1763–1766 (1987).

6. Projan, S. J. *et al.* Comparative sequence and functional analysis of pT181 and pC221, cognate plasmid replicons from *Staphylococcus aureus*. *Mol. Gen. Genet.* **199,** 452–464 (1985).
7. Laddaga, R. A., Chu, L., Misra, T. K. & Silver, S. Nucleotide sequence and expression of the mercurial-resistance operon from *Staphylococcus aureus* plasmid pI258. *Proc. Natl. Acad. Sci. U.S.A.* **84,** 5106–5110 (1987).

a

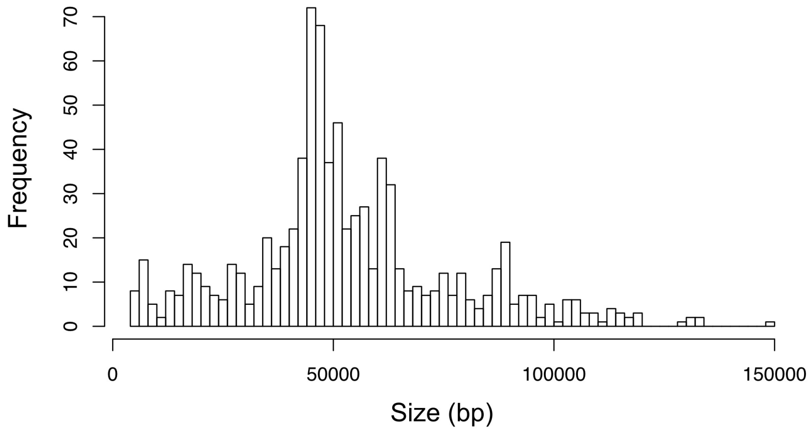

b

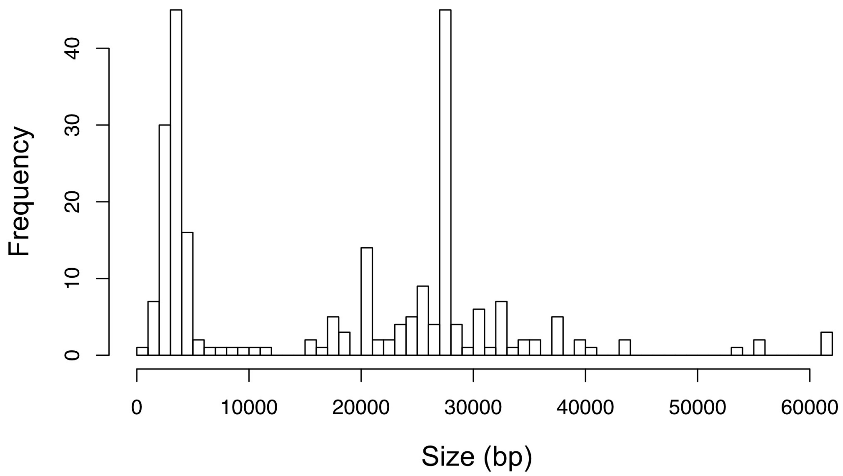

Supplementary Figure 1. Staphylococcal plasmids display bimodal size distribution. 295 complete *S. aureus* sequences were downloaded from the NCBI database and screened for the presence of prophages (n=813) and plasmids (n=243). **(a)** Prophage genomes ranked according to size. **(b)** Plasmid sequences ranked according to size. Almost all plasmids were below 45 kb, which is consistent with phage capsid capacity. Prophages display unimodal distribution with a peak at 44 kb, while plasmid sequences reveal a clear bimodal size distribution with peaks at 4 kb and 27 kb.

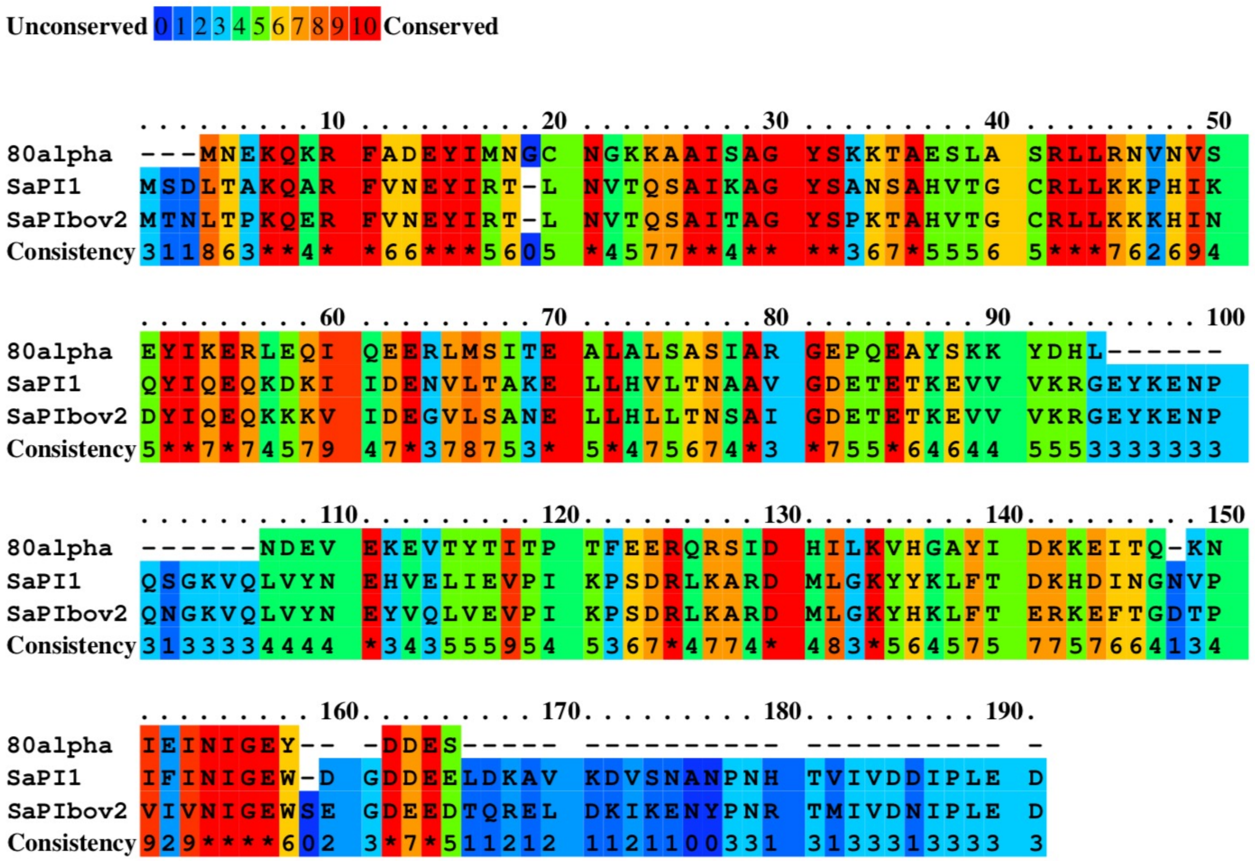

Supplementary Figure 2. Comparison of phage 80α and SaPI small terminase sequences. Small terminase (TerS) amino acid sequences from phage 80α, SaPI1 and SaPIbov2 aligned using PRALINE software.
