# Supplementary material for: Staphylococcal phages and pathogenicity islands drive plasmid evolution
Source: Nat Commun. 2021 Oct 6;12:5845. doi: 10.1038/s41467-021-26101-5 (PMC8494744; doi:10.1038/s41467-021-26101-5)
Supplement: Supplementary file 3 — Description of Additional Supplementary Files [file 41467_2021_26101_MOESM3_ESM.pdf]

Description of Additional Supplementary Files

File Name: Supplementary Data 1
Description: Deleted regions from evolved pGO1 plasmids.

File Name: Supplementary Data 2
Description: Presence of plasmids, SaPIs and prophages in S. aureus.
