# Supplementary material for: Staphylococcal phages and pathogenicity islands drive plasmid evolution
Source: Nat Commun. 2021 Oct 6;12:5845. doi: 10.1038/s41467-021-26101-5 (PMC8494744; doi:10.1038/s41467-021-26101-5)
Supplement: Supplementary file 6 — Reporting Summary [file 41467_2021_26101_MOESM6_ESM.pdf]

nature research

Corresponding author(s): José R. Penadés

Last updated by author(s): Aug 26, 2021

Reporting Summary

Nature Research wishes to improve the reproducibility of the work that we publish. This form provides structure for consistency and transparency in reporting. For further information on Nature Research policies, see Authors & Referees and the Editorial Policy Checklist .

Statistics

For all statistical analyses, confirm that the following items are present in the figure legend, table legend, main text, or Methods section.

n/a	Confirmed	
[ ]	[x]	The exact sample size (*n*) for each experimental group/condition, given as a discrete number and unit of measurement
[ ]	[x]	A statement on whether measurements were taken from distinct samples or whether the same sample was measured repeatedly
[ ]	[x]	The statistical test(s) used AND whether they are one- or two-sided<br>*Only common tests should be described solely by name; describe more complex techniques in the Methods section.*
[x]	[ ]	A description of all covariates tested
[ ]	[x]	A description of any assumptions or corrections, such as tests of normality and adjustment for multiple comparisons
[ ]	[x]	A full description of the statistical parameters including central tendency (e.g. means) or other basic estimates (e.g. regression coefficient) AND variation (e.g. standard deviation) or associated estimates of uncertainty (e.g. confidence intervals)
[ ]	[x]	For null hypothesis testing, the test statistic (e.g. *F*, *t*, *r*) with confidence intervals, effect sizes, degrees of freedom and *P* value noted<br>*Give P values as exact values whenever suitable.*
[x]	[ ]	For Bayesian analysis, information on the choice of priors and Markov chain Monte Carlo settings
[x]	[ ]	For hierarchical and complex designs, identification of the appropriate level for tests and full reporting of outcomes
[x]	[ ]	Estimates of effect sizes (e.g. Cohen's *d*, Pearson's *r*), indicating how they were calculated

Our web collection on statistics for biologists contains articles on many of the points above.

Software and code

Policy information about availability of computer code

Data collection	DNA reference sequences were obtained from NCBI, Genbank
Data analysis	Statistical analyses were performed using GraphPad Prism v9 or R v3.4.2, selecting the appropriate test as indicated in the Materials and Methods as well as in the figure legends within the manuscript.<br>SnapGene Viewer v5.3.1 (https://www.snapgene.com) was used to analyse contigs obtained from pGO1evol, from sequencing and assembly performed by MicrobesNG (https://microbesng.com).<br>DNA alignments were performed using EasyFig v2.2.2 (https://mjsull.github.io/Easyfig/)<br>Protein similarity comparisons were performed using PRALINE web server (http://www.ibi.vu.nl/programs/pralinewww/)<br>The algorithm phiSpy was used to identify prophages in the bacterial chromosomes.<br>CONJScan20, a MacSyFinder module, was used to identify conjugative systems in the 243 plasmids found in S. aureus genomes.

For manuscripts utilizing custom algorithms or software that are central to the research but not yet described in published literature, software must be made available to editors/reviewers. We strongly encourage code deposition in a community repository (e.g. GitHub). See the Nature Research guidelines for submitting code & software for further information.

Data

Policy information about availability of data

All manuscripts must include a data availability statement. This statement should provide the following information, where applicable:

- Accession codes, unique identifiers, or web links for publicly available datasets
- A list of figures that have associated raw data
- A description of any restrictions on data availability

Source data is available in the accompanying excel file.
Accession numbers (obtained from the NCBI database) for reference genomes and the bioinformatics analyses are included in the manuscript or accompanying data files.

Field-specific reporting

Please select the one below that is the best fit for your research. If you are not sure, read the appropriate sections before making your selection.

[x] Life sciences [ ] Behavioural & social sciences [ ] Ecological, evolutionary & environmental sciences

For a reference copy of the document with all sections, see nature.com/documents/nr-reporting-summary-flat.pdf

Life sciences study design

All studies must disclose on these points even when the disclosure is negative.

Sample size	No calculations for determining sample size were required for this study. Experiments were repeated at least 3-times with defined comparison groups: Wild-type against mutant, Presence of SaPI or not, etc. followed by statistical analysis as described. No sample size calculations were required for these direct comparisons.
Data exclusions	No data were excluded from the analysis
Replication	Experiments were performed at least three times as independent biological replicates. Information regarding the number of independent replicates performed are included in the methods section of the manuscript, as well as in the figure legends. Experimental observations were reproducible for all replication attempts.
Randomization	Randomization was not relevant to this study as the genetic elements (i.e. phages, SaPIs, plasmids) that were compared had clearly defined genetic differences.
Blinding	Blinding was not relevant to this study as phenotypes of strains with clearly defined genetic differences (mobile genetic element content or gene mutations) were compared.

Reporting for specific materials, systems and methods

We require information from authors about some types of materials, experimental systems and methods used in many studies. Here, indicate whether each material, system or method listed is relevant to your study. If you are not sure if a list item applies to your research, read the appropriate section before selecting a response.

Materials & experimental systems

n/a	Involved in the study	
[ ]	[x]	Antibodies
[x]	[ ]	Eukaryotic cell lines
[x]	[ ]	Palaeontology
[x]	[ ]	Animals and other organisms
[x]	[ ]	Human research participants
[x]	[ ]	Clinical data

Methods

n/a	Involved in the study	
[x]	[ ]	ChIP-seq
[x]	[ ]	Flow cytometry
[x]	[ ]	MRI-based neuroimaging

Antibodies

Antibodies used	Anti-Digoxigenin-AP, Fab fragments, Roche, product 11093274910
Validation	General validation against specifications, Roche. No further information available.<br>Positive controls were included in every experiment to confirm that all technical steps of the experiment had worked as expected.
